# From Mechanical Cues to Lung Injury: Mapping the Global Landscape and Frontiers of Mechanoregulation Through Bibliometrics and LDA Analysis

**DOI:** 10.1111/jcmm.71377

**Published:** 2026-09-25

**Authors:** Liyan Luo, Bin Yao, Qiqi Ruan, Yu He, Meiyu Zhang, Qing Ai, Qiuju Wu, Sile Hu, Ying Zhang, Feng Jiang, Yuan Shi

**Affiliations:** ^1^ Department of Neonatology Children's Hospital of Chongqing Medical University, National Clinical Research Center for Children and Adolescents's Health and Disorders, Ministry of Education Key Laboratory of Child Development and Disorders, Chongqing Key Laboratory of Child Rare Diseases in Infection and Immunity Chongqing China; ^2^ Department of Neonatology Dali Bai Autonomous Prefecture Maternal and Child Health Care Hospital Dali China; ^3^ Department of Neonatology Affiliated Hospital of Inner Mongolia Medical University Huhehaote China; ^4^ Department of Neonatology Xiangyang No. 1 People's Hospital, Hubei University of Medicine Xiangyang China; ^5^ Department of Neonatology Obstetrics & Gynecology Hospital of Fudan University, Shanghai Key Lab of Reproduction and Development, Shanghai Key Lab of Female Reproductive Endocrine Related Diseases Shanghai China

**Keywords:** bibliometric analysis, CiteSpace, lung injury, matrix stiffness, mechano‐immunology, mechanotransduction

## Abstract

Pulmonary cells continuously experience mechanical forces, yet the rapid expansion of mechanotransduction research has fragmented the knowledge base and obscured how research priorities have shifted over time. We therefore mapped the intellectual structure and thematic evolution of pulmonary mechanoregulation using bibliometric analysis and latent Dirichlet allocation (LDA). A total of 1896 articles and reviews published during 2000–2025 were retrieved from the Web of Science Core Collection and analysed with CiteSpace and LDA. Annual output increased from fewer than 60 publications per year before 2012 to more than 160 in 2024. Citation, co‐occurrence, clustering and burst analyses showed a transition from macroscopic injury paradigms, including cyclic stretch and alveolar overdistension, towards matrix stiffness. Yes‐associated protein/transcriptional coactivator with PDZ‐binding motif (YAP/TAZ) signalling, mechanosensitive ion channels and mechano‐immunology. LDA resolved 15 latent topics; idiopathic pulmonary fibrosis and macrophage activation was the largest topic (254 documents), while temporal modelling showed increasing prominence of macrophage‐centred fibrosis and viral‐associated lung injury and declining emphasis on classical mechanical stretch. These findings define major knowledge gaps at the biomechanics–immunology interface and provide a quantitative roadmap for prioritizing biomimetic models, multi‐omics integration and selective mechanotherapeutic strategies.

## 1 Introduction

The human lung is a highly dynamic organ that operates under continuous mechanical stimulation [1, 2]. During each respiratory cycle, alveolar tissues experience repeated deformation from cyclic stretch, shear stress and surface tension [3]. Under physiological conditions, these mechanical cues are essential for tissue homeostasis, surfactant secretion and maintenance of the alveolar‐capillary barrier [4, 5]. In pathological states, however, the lung's mechanical environment can become profoundly disrupted [6]. For example, progressive stiffening of the extracellular matrix occurs during pulmonary fibrosis [7, 8], while mechanical ventilation, though life‐saving in acute respiratory distress syndrome (ARDS), can impose excessive forces on vulnerable lung tissue, leading to ventilator‐induced lung injury (VILI) [9, 10]. This underscores the dual nature of mechanical forces: essential for normal function yet capable of driving cellular injury and organ failure when dysregulated [11]. Understanding cellular sensing and responses to these forces, termed mechanotransduction, is therefore critical to elucidating the pathogenesis of severe lung diseases [12, 13].

Over the past two decades, mechanotransduction research has progressed from macroscopic observations to detailed molecular mechanisms. Early studies focused on the harmful effects of high tidal volumes and airway pressures, establishing the principles of lung‐protective ventilation [14]. More recently, attention has shifted to the cellular microenvironment, where cells sense extracellular matrix stiffness through integrins and mechanosensitive ion channels, including Piezo‐type mechanosensitive ion channel component 1 (PIEZO1) and transient receptor potential vanilloid 4 (TRPV4) [15, 16]. These mechanical inputs converge on intracellular effectors such as Yes‐associated protein (YAP), transcriptional coactivator with PDZ‐binding motif (TAZ) and Rho‐associated coiled‐coil‐containing protein kinase (ROCK), promoting fibroblast activation, epithelial–mesenchymal transition and dysregulated immune responses [17, 18]. This shift represents a paradigm change, suggesting that targeting mechanoregulatory checkpoints could yield novel therapies for treatment‐resistant lung diseases beyond supportive care alone [19].

Fuelled by these advances, the literature on pulmonary mechanoregulation has expanded exponentially, becoming highly interdisciplinary by integrating biomechanics, biophysics, molecular biology and critical care medicine [20, 21]. In the literature corpus analysed in the present study, annual publication output remained below 60 articles per year before 2012 but exceeded 160 publications in 2024, illustrating both the rapid expansion and diversification of this field. Tracking temporal shifts in research hotspots is therefore important not merely for describing publication growth but also for distinguishing mature topics from emerging mechanisms, identifying underexplored links between mechanical cues and specific pulmonary diseases and guiding future experimental and translational priorities. Traditional systematic reviews can synthesize individual subtopics but are less suited to reconstructing large‐scale chronological shifts, collaborative structures and latent thematic relationships [22]. Without such a temporal knowledge map, rapidly emerging areas such as matrix stiffness, mechanosensitive ion channels and mechano‐immunology may remain obscured within an increasingly fragmented literature.

Bibliometric analysis provides an objective, quantitative approach to address these limitations. Using the Web of Science Core Collection (WoSCC) and CiteSpace software, this study delivers a high‐resolution mapping of the research landscape across the calendar years 2000–2025, serving as both a statistical overview and a strategic guide. Specifically, we pursued three objectives: (1) quantify global publication trends and identify dominant countries, institutions and collaborative networks; (2) trace thematic evolution from macroscopic mechanical stress to molecular mechanotransduction mechanisms, supplemented by latent Dirichlet allocation (LDA) topic modelling to uncover hidden semantic structures within abstracts through probabilistic modelling [23]; and (3) detect citation bursts to pinpoint the most active research frontiers. We hypothesize that future breakthroughs will arise from integrating high‐throughput omics, elucidating mechano‐immune crosstalk and developing precision mechanotherapeutics. By synthesizing these analyses, this study offers a roadmap for navigating the field's complex history and promising future directions.

## 2 Materials and Methods

### 2.1 Data Sources and Search Strategy

Bibliographic records were retrieved from the WoSCC, specifically the Science Citation Index Expanded (SCI‐E). To minimize bias from daily database updates, all searches and data downloads were completed on a single day (10 January 2026).

The search strategy targeted publications on mechanotransduction in lung injury using the following query: TS = (((‘acute lung injury’ OR ALI OR ARDS OR VILI OR ‘VILI’ OR fibrosis OR ‘pulmonary fibrosis’ OR ‘lung fibrosis’ OR ‘barrier dysfunction’) AND (lung OR pulmonary)) AND (mechanotransduction OR mechanosens* OR ‘cyclic stretch’ OR ‘shear stress’ OR ‘matrix stiffness’ OR integrin* OR YAP OR TAZ OR ROCK OR PIEZO1 OR TRPV4)). Pharmacokinetic terms were not included as independent search parameters because the primary objective of this study was to characterize mechanotransduction and mechanoregulation in pulmonary injury rather than drug absorption, distribution, metabolism or elimination. Inclusion of broad pharmacokinetic terminology could substantially reduce search specificity by retrieving drug‐disposition studies unrelated to mechanical signalling. Importantly, pharmacological and therapeutic studies were not excluded and remained eligible when they also contained the predefined lung‐injury and mechanotransduction terms. The publication period was restricted to 2000–2025.

### 2.2 Inclusion and Exclusion Criteria

**FIGURE 1:**
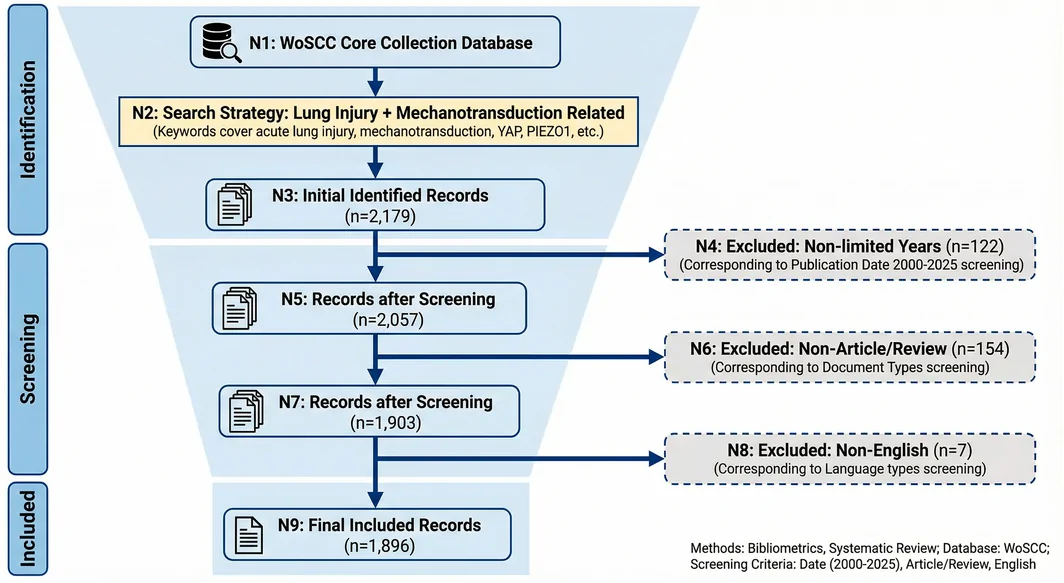
Flowchart of the literature search and screening process.

Records were included if they met the following criteria: (1) published between 1 January 2000 and 31 December 2025; (2) document type classified as ‘Article’ or ‘Review’ and (3) written in English. Editorials, meeting abstracts, letters and corrections were excluded. The screening process is shown in Figure 1. After screening, 1896 valid records were exported in plain‐text format (Full Record and Cited References) for analysis.

### 2.3 Bibliometric Analysis and Visualization

CiteSpace (version 6.4.R2, Drexel University, USA), a Java‐based visualization tool, was used to map knowledge domains and detect structural patterns and evolutionary trends in the field [24]. Key parameters were configured as follows: Time slicing: 1‐year intervals from 2000 to 2025. Node types: Country, Institution, Author, Cited Author, Cited Journal, Reference and Keyword (selected individually for specific analyses). Selection criteria: Top 50 most cited or frequent items per time slice. Pruning: The Pathfinder algorithm and pruning of sliced networks were applied to simplify networks and improve readability.

### 2.4 Data Interpretation Metrics

Three core bibliometric indicators were evaluated: Betweenness centrality: Nodes with centrality > 0.1 were considered pivotal hubs or turning points in the network. Clustering: Keywords and references were clustered using the log‐likelihood ratio (LLR) method. Cluster quality was assessed by Modularity *Q* (*Q* > 0.3 indicates meaningful structure) and Mean Silhouette score (*S* > 0.7 indicates high cluster consistency). Burst detection: Kleinberg's algorithm identified terms or references with sudden citation surges, marking emerging hotspots or frontiers. A dual‐map overlay of journals was also generated to illustrate citation trajectories and interdisciplinary connections between citing and cited journals.

### 2.5 LDA Topic Modelling

To uncover latent semantic structures beyond citation networks, LDA topic modelling was applied [25]. Titles and abstracts of the 1896 included publications formed the textual corpus. Preprocessing included tokenization, removal of standard English stop words, punctuation and numbers, followed by stemming to normalize word forms. A document‐term matrix was constructed to represent term frequencies. The LDA generative model was then used to extract latent topics based on word co‐occurrence patterns. The optimal number of topics was selected by maximizing model coherence and interpretability. Annual average topic probabilities were calculated and fitted with linear regression to assess temporal trends. Principal component analysis (PCA) was applied to the topic‐document probability matrix to visualize semantic relationships and clustering among topics.

## 3 Results

Using CiteSpace‐based bibliometric mapping together with LDA topic modelling, we analysed 1896 articles and reviews on pulmonary mechanoregulation and mechanotransduction in lung injury and disease published during the calendar years 2000–2025. The analyses characterized publication growth, collaborative networks, intellectual structure, research hotspots, citation bursts and temporal evolution of latent semantic topics.

### 3.1 Literature Search and Screening Process

The record selection procedure is outlined in Figure 1. The initial search of the WoSCC yielded 2179 records. After applying inclusion and exclusion criteria, 122 publications were removed for falling outside the 2000–2025 timeframe, 154 were excluded due to document type (non‐article/review) and 7 non‐English entries were eliminated. Ultimately, 1896 eligible publications were retained for bibliometric analysis.

### 3.2 Annual Publication Growth and Trends

**FIGURE 2:**
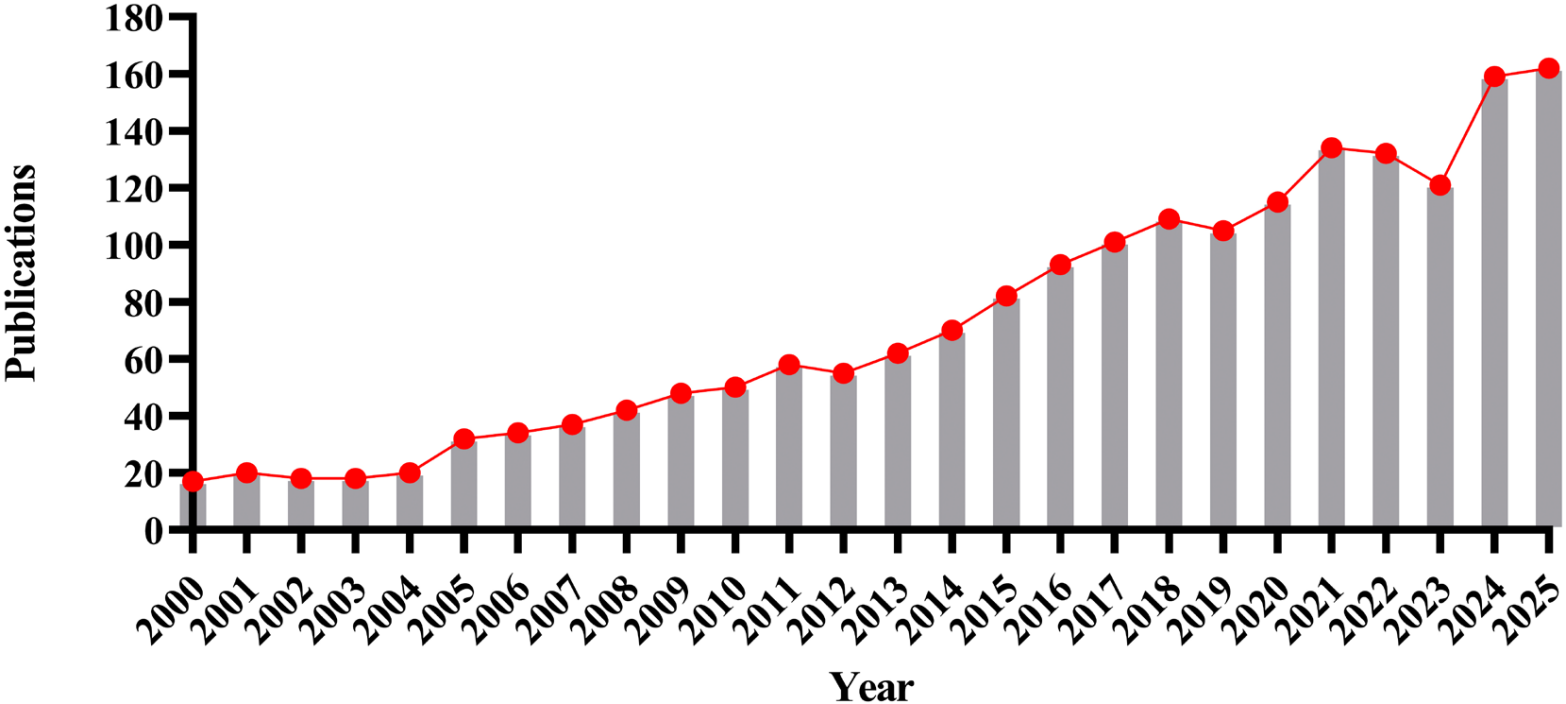
Annual publication trends.

Annual publication output is shown in Figure 2. Over the 25 years, the field exhibited steady growth in scientific productivity, reflecting rising interest in pulmonary mechanoregulation. From 2000 to 2011, annual publications remained below 60. A marked inflection occurred around 2012, after which output accelerated rapidly, peaking at over 160 publications in 2024. This trajectory underscores the increasing centrality of mechanical cues in pulmonary research.

### 3.3 Global Geographic Distribution and Collaboration

**FIGURE 3:**
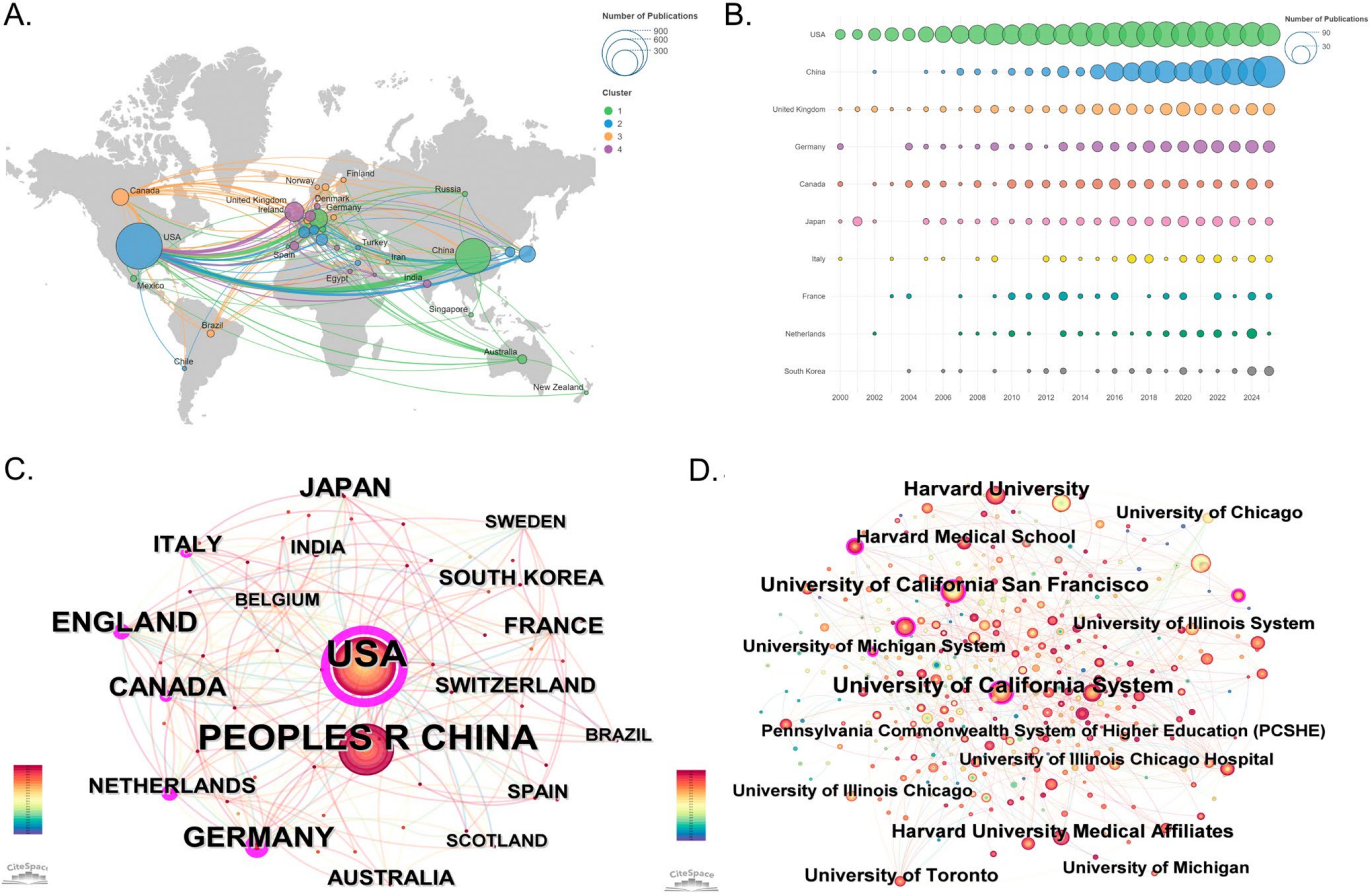
Global geographic distribution and institutional collaboration networks. (A) World map illustrating the geographic density of publications. (B) The temporal evolution of publication output for the top 10 most productive countries. (C) Country co‐occurrence network analysis. The size of the nodes represents the total number of publications, and the connecting lines indicate collaborative links. (D) Institutional collaboration network.

The geographic distribution of research contributions is depicted in Figure 3. Publications were concentrated in North America, East Asia and Europe (Figure 3A). Among the top 10 most productive countries (Figure 3B and Table S1), the United States maintained consistent leadership throughout the period, while China displayed explosive growth after 2012, substantially closing the gap. The country co‐occurrence network (Figure 3C) highlights this bipolar dominance, with the United States and China as the largest nodes. Link thickness reveals strong international collaboration, particularly involving the United States, European countries (e.g., England and Germany) and Asian nations.

### 3.4 Institutional Contributions and Cooperation Networks

Institutional collaboration is illustrated in Figure 3D and Table S1. US institutions dominated, forming dense cooperative clusters. The University of California System (including UCSF) and Harvard University occupied central network positions as key hubs for knowledge dissemination. Other prominent contributors included the University of Chicago, the University of Illinois System and the University of Michigan. The network structure indicates a mature, highly interconnected research community driven primarily by US‐based institutions with extensive cross‐institutional partnerships.

### 3.5 Analysis of Influential Authors and Co‐Authorship Networks

**FIGURE 4:**
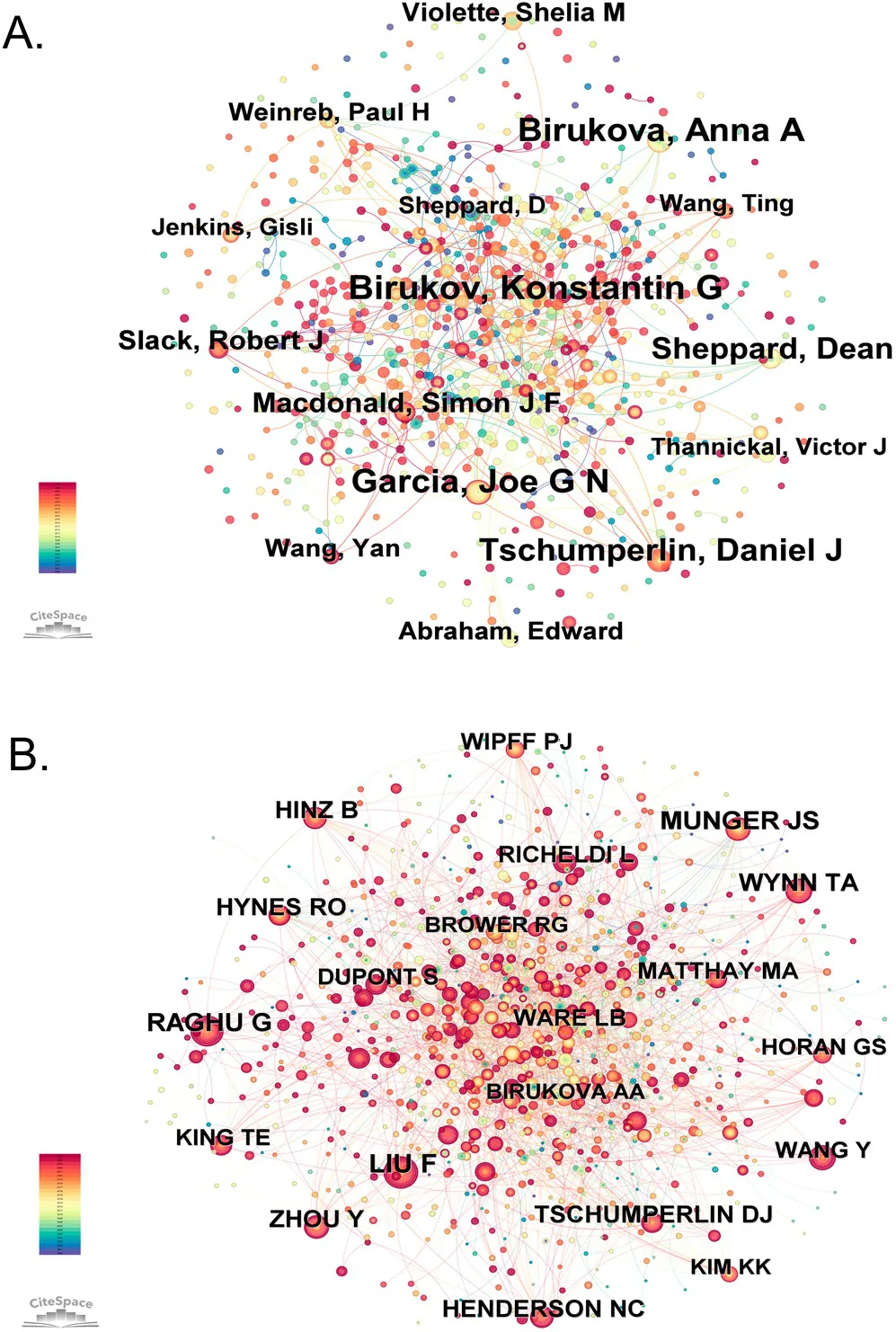
Author contribution and influence analysis. (A) Author co‐occurrence network showing distinct collaborative clusters among prolific researchers. (B) Author co‐citation network visualizing the intellectual structure of the field. Node size is proportional to the citation frequency.

The author co‐occurrence network (Figure 4A and Table S2) revealed distinct collaborative clusters, with prolific researchers such as K.G. Birukov, A.A. Birukova and J.G.N. Garcia forming closely connected groups in pulmonary mechanobiology. The author co‐citation network (Figure 4B and Table S2) further identified foundational intellectual influences. L.B. Ware and M. A. Matthay emerged as central nodes, consistent with their landmark 2000 synthesis of ARDS pathophysiology [26]. S. Dupont became highly influential following the 2011 identification of YAP/TAZ as nuclear mediators of mechanotransduction, whereas D.J. Tschumperlin contributed key evidence in 2010 demonstrating that matrix stiffening can actively amplify pulmonary fibrosis rather than merely represent its consequence [27, 28]. Together, these highly co‐cited contributions illustrate the convergence of clinical lung injury research with fundamental mechanobiology.

### 3.6 Journal Distribution and Interdisciplinary Knowledge Flow

**FIGURE 5:**
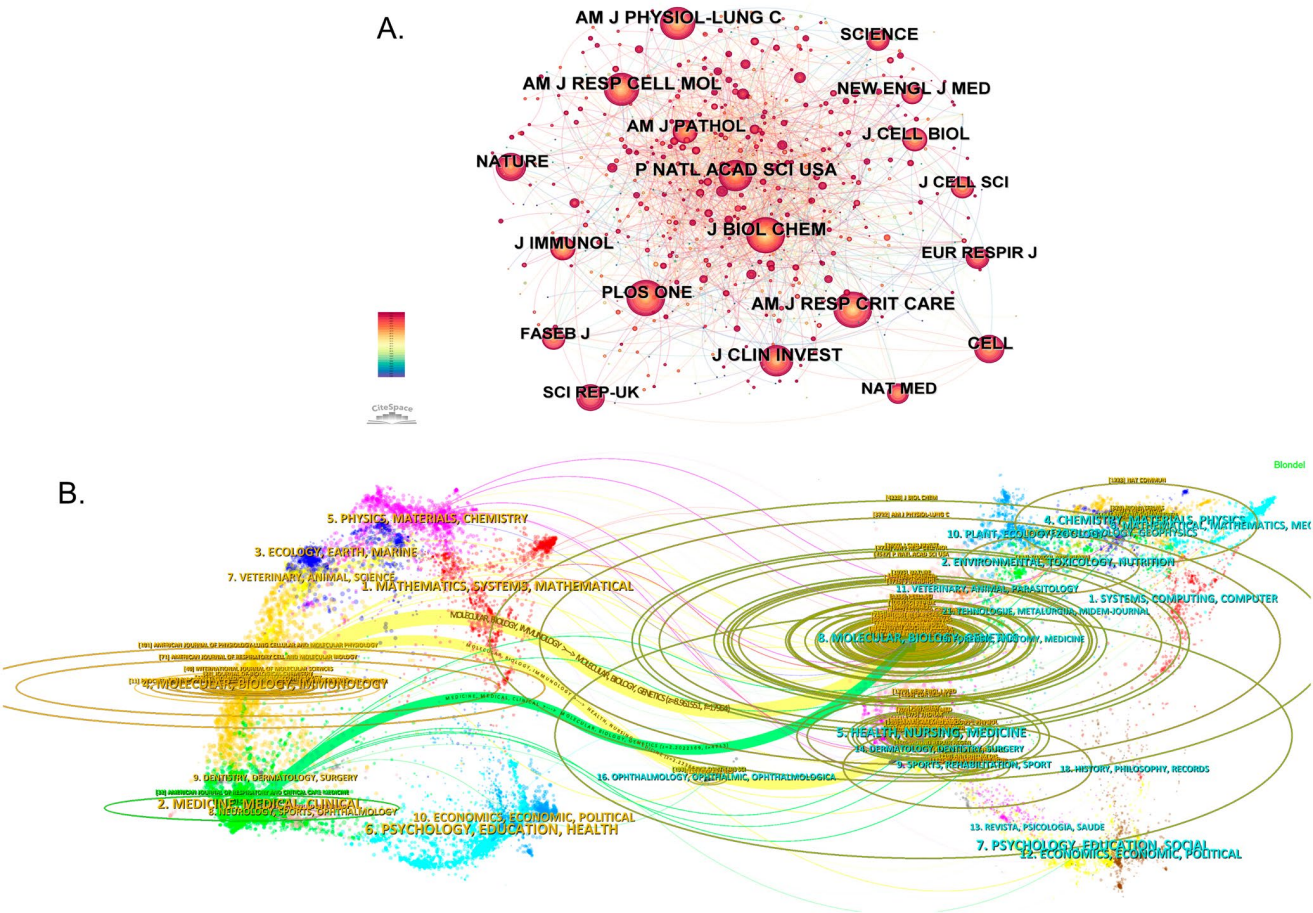
Journal distribution and interdisciplinary knowledge flow. (A) Journal co‐citation network showing the core sources of knowledge, primarily centred on respiratory medicine and cell biology. (B) Dual‐map overlay of journals. The left side represents citing journals (research frontiers) and the right side represents cited journals (knowledge base). The coloured paths illustrate the citation trajectory, indicating that clinical/physiological studies (left) heavily rely on foundational discoveries in molecular genetics and biology (right).

The journal co‐citation network (Figure 5A and Table S3) identifies core knowledge sources. High‐impact journals in respiratory medicine (e.g., *American Journal of Respiratory and Critical Care Medicine* and *American Journal of Physiology‐Lung Cellular and Molecular Physiology*) and cell biology (e.g., *Journal of Biological Chemistry*, *Journal of Cell Biology* and *Nature*) formed the network core. This pattern reflects synergy between clinical physiology and molecular mechanisms. The dual‐map overlay (Figure 5B) illustrates citation flows across disciplines. The primary trajectory originated from journals in the *Molecular, Biology, Immunology* and *Medicine, Medical, Clinical* domains (left side), which predominantly cited foundational work in *Molecular, Biology, Genetics* journals (right side). This indicates that clinical and physiological studies of lung injury relied heavily on advances in molecular and cellular biology.

### 3.7 Thematic Evolution and Knowledge Base Clustering

**FIGURE 6:**
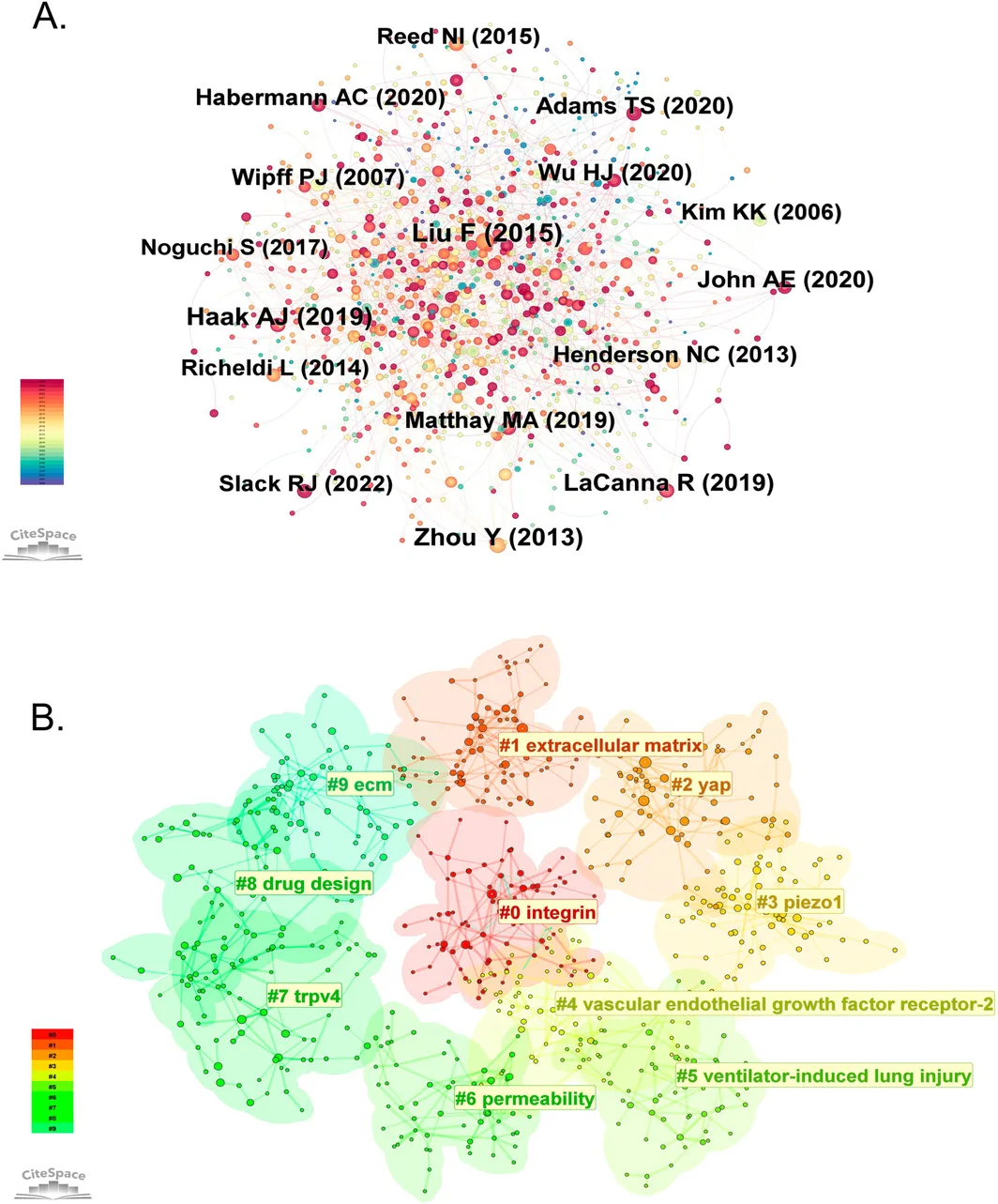
Thematic evolution and reference analysis. (A) Reference co‐citation network. (B) Cluster analysis of co‐cited references.

The reference co‐citation network (Figure 6A and Table S4) and its clusters (Figure 6B) reveal the field's thematic progression. Major clusters, labelled by LLR, included #0 integrin, #1 extracellular matrix, #2 YAP and #3 PIEZO1. This structure traces a conceptual evolution: early focus on structural injury mechanisms (e.g., #5 VILI and #6 permeability) shifted towards extracellular matrix remodelling (#1) and transmembrane signalling via integrins (#0), culminating in specific mechanotransducers and effectors such as YAP (#2) and PIEZO1 (#3).

Citation burst analysis of the top 25 references (Figure S1) pinpointed active frontiers. Early bursts (2000–2015) featured seminal works linking myofibroblast contraction to latent TGF‐β activation (e.g., P.J. Wipff 2007; K.K. Kim 2006). Mid‐period bursts (2013–2018) emphasized matrix stiffness and integrin‐mediated fibrosis (e.g., Y. Zhou 2013; N.C. Henderson 2013). Recent and ongoing bursts (2019–2025) highlighted single‐cell RNA sequencing for mapping fibrotic niches, specific cell‐type roles (e.g., fibroblasts and alveolar epithelial cells) and targeted therapies (e.g., A.J. Haak 2019; H.J. Wu 2020; R.J. Slack 2022). These trends underscore the rise of high‐throughput omics and precision mechanotherapeutics as current frontiers.

### 3.8 Keyword Co‐Occurrence and Thematic Clustering

**FIGURE 7:**
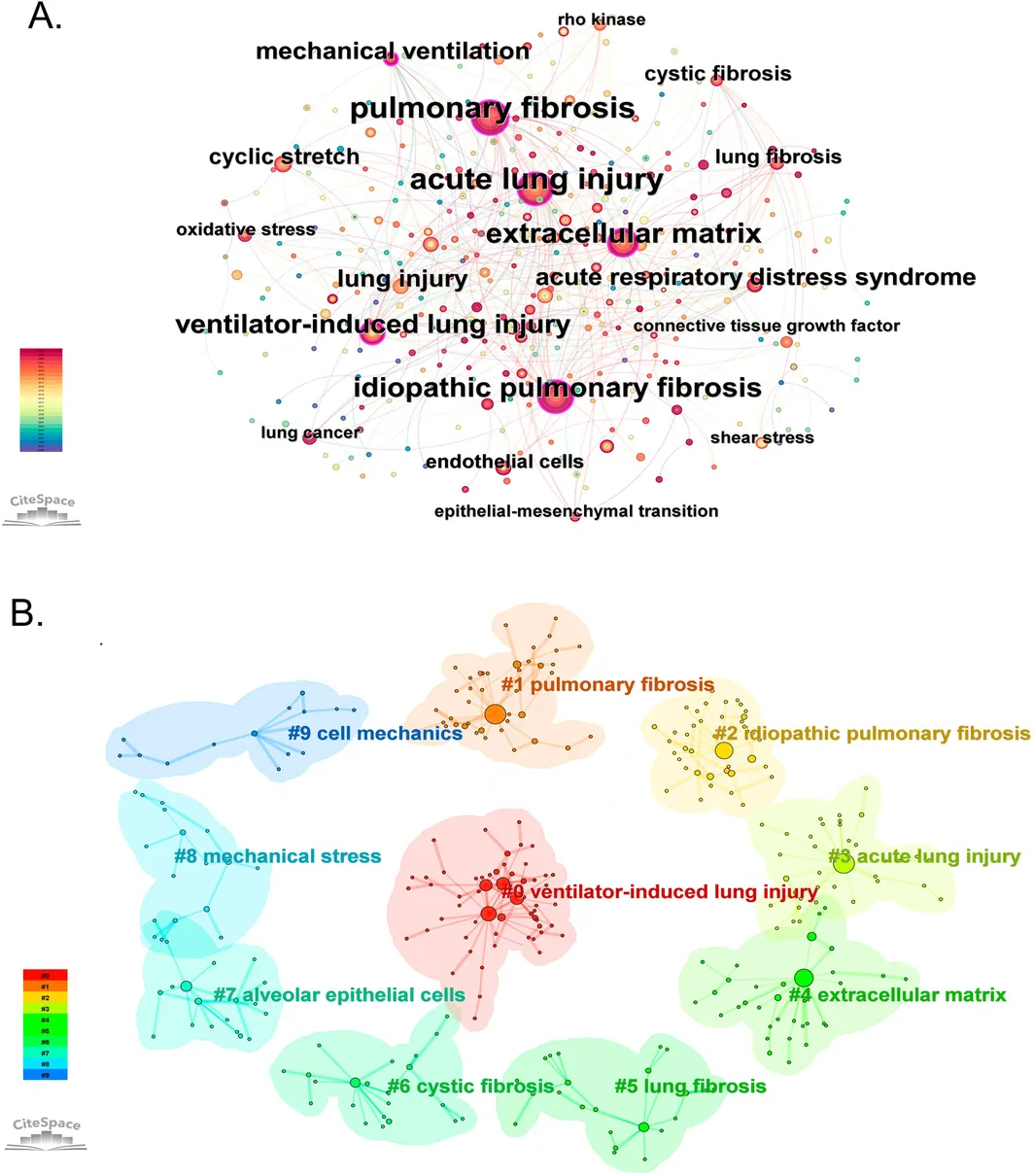
Keyword co‐occurrence and thematic clustering. (A) Network map of high‐frequency keywords. The node size corresponds to the frequency of occurrence. (B) Cluster view of the keyword network, dividing the field into 10 distinct topics ranging from disease phenotypes.

The keyword co‐occurrence network (Figure 7A) delineates the field's conceptual framework, anchored by high‐frequency terms such as ‘pulmonary fibrosis’, ‘acute lung injury’, ‘extracellular matrix’ and ‘ventilator‐induced lung injury’. Peripheral nodes like ‘epithelial‐mesenchymal transition’ and ‘endothelial cells’ linked mechanical forces to disease phenotypes. Cluster analysis (Figure 7B) identified 10 distinct groups, spanning disease models (e.g., #0 ventilator‐induced lung injury and #2 idiopathic pulmonary fibrosis) to cellular responses (e.g., #7 alveolar epithelial cells and #9 cell mechanics). High network modularity confirmed a structured yet interconnected knowledge base.

**FIGURE 8:**
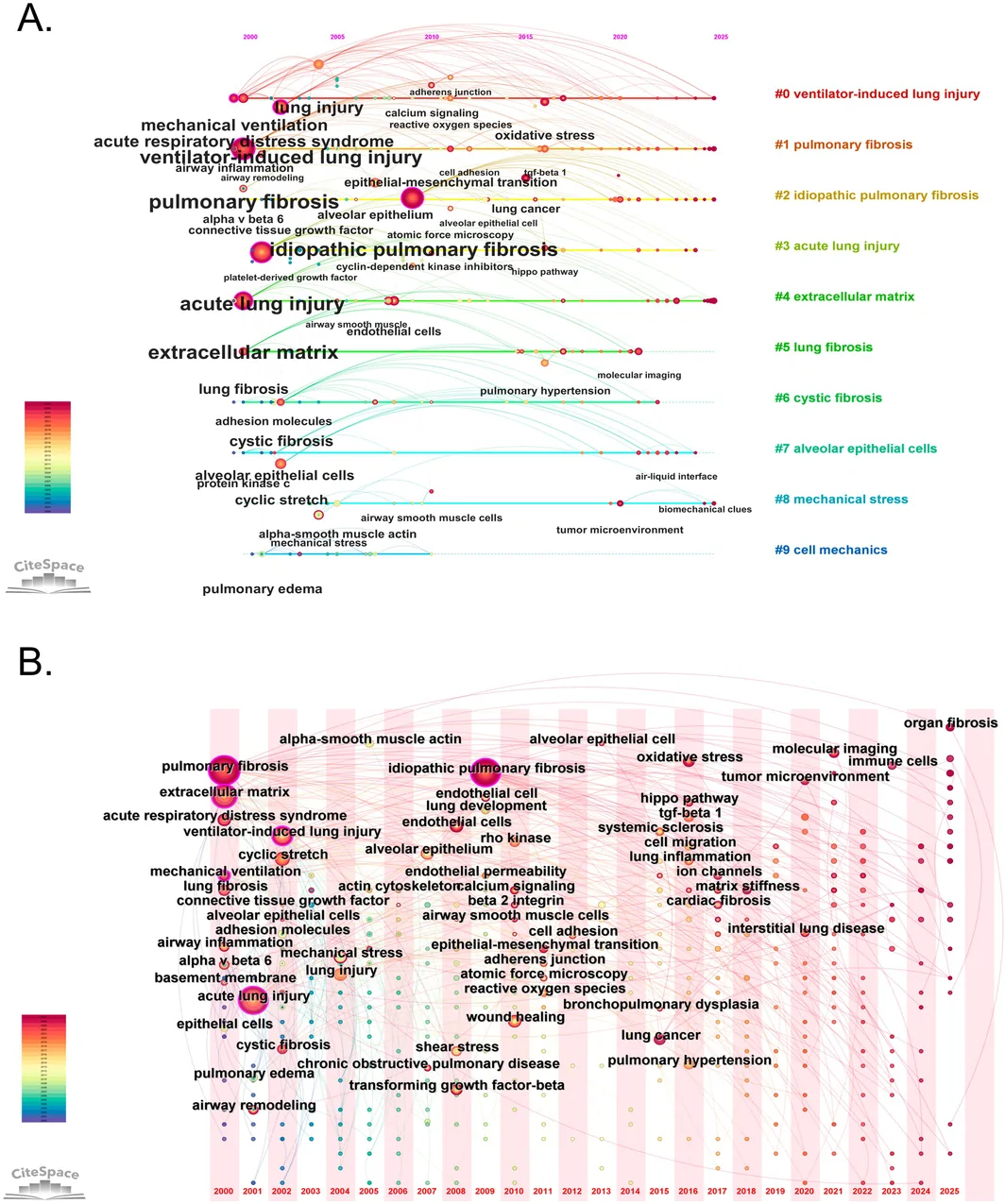
Chronological evolution of research hotspots. (A) Timeline view of keyword clusters, visualizing the lifespan and evolution of specific sub‐topics. (B) Timezone view illustrating the transition of research focus over three phases: from macroscopic ventilation strategies (Phase I) to molecular mechanotransduction pathways (Phase II) and, more recently, to precision medicine and micro‐environmental cues (Phase III).

Timeline and timezone views (Figure 8A,B) traced hotspot evolution. Phase I (2000–2010) emphasized macroscopic triggers and syndromes (e.g., ‘mechanical ventilation’, ‘cyclic stretch’, ‘acute respiratory distress syndrome’; clusters #0 and #3). Phase II (2011–2018) shifted to cellular and molecular pathways (e.g., ‘epithelial‐mesenchymal transition’, ‘TGF‐β1’ and ‘connective tissue growth factor’). Phase III (2019–2025) diversified into microenvironmental cues and precision approaches (e.g., ‘matrix stiffness’, ‘Hippo pathway’ and ‘tumour microenvironment’).

Keyword burst detection (Figure S2) provided robust evidence of emerging frontiers among the top 25 terms. Early bursts centred on ‘cyclic stretch’ (2015–2019), superseded by ‘matrix stiffness’ and ‘ion channels’ (2023–2025), signalling a transition from external loading to intrinsic tissue properties and mechanosensitive channels (e.g., PIEZO1 and TRPV4). ‘Immune cells’ showed strong ongoing bursts (2023–2025), indicating rising interest in mechano‐immune crosstalk. Additional recent bursts included ‘air–liquid interface’ (reflecting advanced in vitro models) and ‘oxidative stress’ (downstream signalling effects).

### 3.9 Semantic Discovery and Topic Evolution via LDA

To gain deeper semantic insight into the corpus beyond keyword co‐occurrence, LDA topic modelling was applied. As summarized in Table 1, the analysis identified 15 distinct latent topics, grouping research into key pillars: fundamental fibrosis mechanisms (Topics 1, 2 and 8), mechanical stress models (Topics 6, 10 and 12), signalling pathways (Topic 3: TGF‐β; Topic 7: NF‐κB) and clinical/viral aetiologies (Topics 4 and 14). Topic 1 (idiopathic pulmonary fibrosis and macrophage activation) was the most prevalent (*N* = 254 documents), highlighting the central role of immune cells in pulmonary mechanopathologies.

**TABLE 1:** Topics discovered from 1896 articles published between 2000 and 2025.

Topic	Prevalence	Top terms	Label	*N*
Topic 1	0.0666	american_physiological; analyse; cells_become; cells_levels; disease_idiopathic; ehp; extend; lungs_exposed; m_macrophage; maards	Idiopathic pulmonary fibrosis and macrophage activation	254
Topic 2	0.0665	advantages; chondroitin; direct_binding; dishes; fibrosis_processes; fibrotic_gene; improve_understanding; lake_aerosols; loaded; lungs_characterized	Pulmonary fibrosis mechanisms and gene expression	198
Topic 3	0.0668	antitgf_beta; blot_immunohistochemistry; cord; cut; desmps; downregulated_proteins; ebola; effects_tgfbeta; fibroblasts_tgf; ilinduced	TGF‐β signalling in pulmonary fibroblasts	184
Topic 4	0.0669	ards_n; ba_bcells; beta_pathway; coronavirus_sarscov; crgdfk; cs_preconditioning; endotoxemia; eventfree_survival; expressed_proteins; fibrosis_previously	ARDS and viral lung injury (SARS‐CoV)	145
Topic 5	0.0668	alkbh; apl_cells; atg; capabilities; cat; compound_b; cpa; endothelial_inflammation; expression_vecadherin; farinaeinduced	Endothelial inflammation and barrier dysfunction	155
Topic 6	0.0672	aggressiveness; ards_vili; breaks; cardiac_dysfunction; cells_generated; contractioninduced; dense_fibrosis; early_passage; ec_exposed; fak_paxillin	Ventilator‐induced lung injury and fibrosis	129
Topic 7	0.0664	antiplatelet; arginineglycineaspartic_acid; associate; barrier_resistance; combine; emerged_key; expression_mouse; factor_kappa; healing_process; ilk_expression	NF‐κB activation and epithelial barrier healing	99
Topic 8	0.0665	activation_nfkappa; alp; arterial_endothelial; assisted; bleomycininduced_pf; completely_inhibited; detecting; exhibited_decreased; heterozygous; latrunculin	Bleomycin‐induced pulmonary fibrosis model	100
Topic 9	0.0665	aav; air_flow; beta_treatment; description; dry_powder; grown_collagen; injury_resolution; injury_review; ino; inspiration	Inhaled therapies and lung injury resolution	106
Topic 10	0.0667	activated_cell; activation_inhibition; barotrauma; brdi; cells_increase; cells_seeded; channels_lung; combined_effects; cspreconditioning; definitive	Mechanical stretch and alveolar cell activation	92
Topic 11	0.0664	adaptive_immunity; airways_alveoli; aspiration_pneumonia; associated_activation; associated_higher; caspases; cellular_uptake; contractile_migratory; define_role; drug_screening	Aspiration pneumonia and adaptive immunity	75
Topic 12	0.0664	activation_collagen; active_form; alveolar_overdistension; calu_cells; cxc; damage_alveolar; direct_role; dmf; duoxa; e_coli	Alveolar overdistension and epithelial injury	79
Topic 13	0.0668	altered_mechanical; chemotherapy; crb; cutting_rock; dapk_expression; diminishes; edta; exclusive; extravasation_lung; food	Chemotherapy‐induced lung toxicity	88
Topic 14	0.0667	admission; adult_respiratory; anaemic; anaemic_thrombocytopenic; appreciation; arg; beta_ilkpik; blood_loss; bt_site; built	ARDS in critically ill and anaemic patients	76
Topic 15	0.0667	apoptosis_inflammation; bleomycin_ad; channels_trpv; colassg; come; coronary; deprivation; differentially_methylated; function_associated; gamma_cinduced	Apoptosis and inflammation in bleomycin injury	75

**FIGURE 9:**
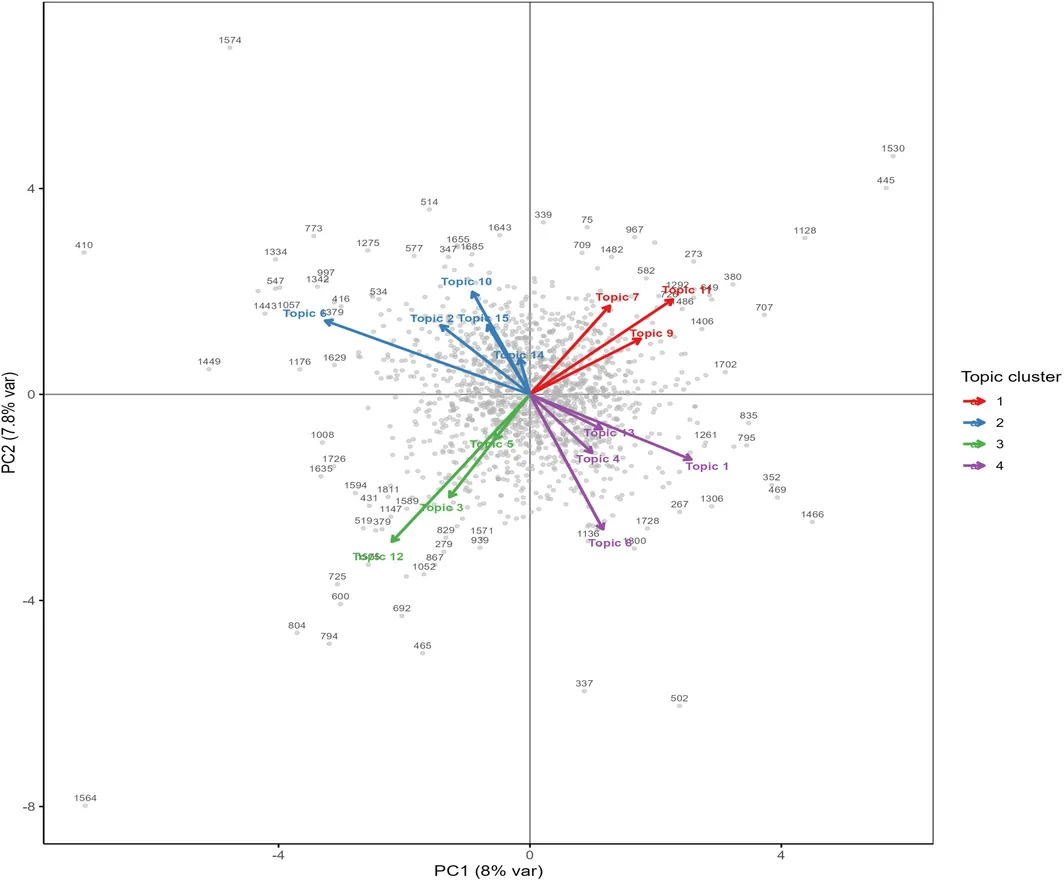
Semantic clustering and inter‐topic relationships visualized via PCA.

Semantic relationships among topics are visualized in the PCA biplot (Figure 9). Classical mechanical injury topics, such as Topic 12 (alveolar overdistension) and Topic 3 (TGF‐β signalling), clustered in the lower‐left quadrant, representing the field's foundational base. In contrast, Topic 1 (macrophage activation) and Topic 4 (ARDS and viral lung injury) projected into the right quadrants, indicating distinct sub‐domains of mechano‐immunology and viral‐induced injury that diverge from traditional sterile mechanical damage.

**FIGURE 10:**
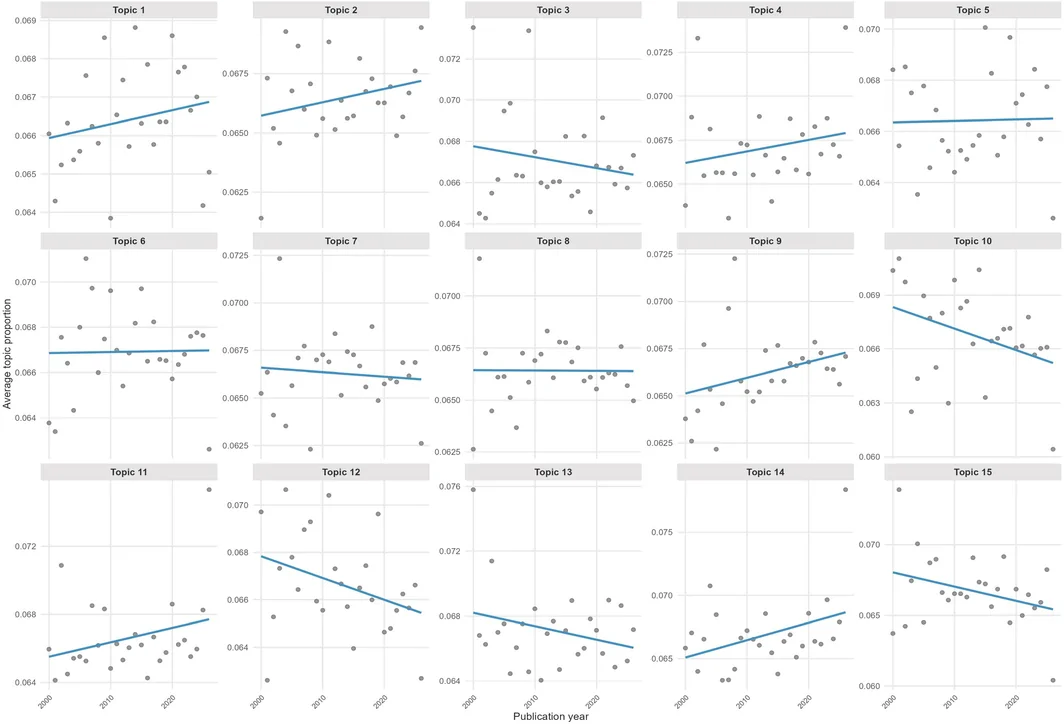
Temporal evolution trends of the 15 identified latent research topics.

Temporal trends in topic prevalence (Figure 10) further confirmed shifting priorities. Traditional physical mechanics topics, including Topic 12 (alveolar overdistension) and Topic 13 (chemotherapy‐induced injury), showed declining or stable trajectories over the past decade. Conversely, topics incorporating cellular complexity exhibited marked increases: Topic 1 (IPF and macrophage activation) rose steeply after 2015, reflecting growing emphasis on biomechanics–immunology intersections; Topic 4 (ARDS and SARS‐CoV‐2) spiked sharply in recent years, driven by integration of mechanotransduction into viral ARDS research (e.g., COVID‐19); and Topic 2 (fibrosis mechanisms and gene expression) increased steadily, aligned with rising use of transcriptomics.

## 4 Discussion

This study presents the first comprehensive bibliometric analysis of global research on mechanotransduction in pulmonary injury and disease over the past 25 years. Our findings confirm exponential growth in publications, particularly after 2012, indicating that pulmonary mechanobiology has evolved from a specialized biophysical niche to a cornerstone of modern respiratory medicine. The principal contribution of this work is the precise mapping of a paradigm shift: research has progressed from macroscopic descriptions of VILI and barotrauma to molecular interrogation of microenvironmental cues, with matrix stiffness, YAP/TAZ signalling and mechanosensitive ion channels (e.g., PIEZO1) emerging as central nodes [29]. This evolution reflects a maturing consensus that mechanical forces are not merely extrinsic stressors but intrinsic drivers of cell fate, tissue remodelling and chronic disease progression.

Clustering and burst analyses illuminate the dynamic lifecycle of knowledge in the field. Early dominance of concepts such as ‘cyclic stretch’, ‘barrier dysfunction’ and ‘alveolar overdistension’ aligned with landmark clinical trials that established lung‐protective ventilation and reduced iatrogenic injury [30]. Yet persistent high mortality in ARDS and progressive fibrosis despite optimized ventilation prompted deeper mechanistic inquiry. Seminal discoveries linking ECM stiffness to latent TGF‐β activation and myofibroblast differentiation via integrins and focal adhesion kinase catalysed this transition [31, 32]. The subsequent identification of YAP/TAZ as master transcriptional effectors provided a unifying framework: physical cues from the stiffened matrix directly reprogram gene expression, sustaining fibrosis long after the inciting insult resolves [33, 34]. This molecular reframing has transformed clinical perception of the lung as a highly mechanosensitive organ in which tissue compliance is itself pathogenic.

Methodological maturation is evident in recent hotspots. Early in vitro studies frequently employed cells cultured on rigid plastic substrates (> 1 GPa stiffness), which artifactually activated profibrotic pathways and poorly recapitulated the compliant physiological lung (~0.5–5 kPa) [35]. The pronounced burst in ‘air–liquid interface’ models, lung‐on‐a‐chip platforms and viscoelastic hydrogels signals recognition of these limitations. Advanced biomimetic systems now replicate breathing‐induced cyclic strain, fluid shear and tunable matrix mechanics, revealing that physiologic deformation alone can induce endothelial leakage or epithelial remodelling independent of inflammatory mediators [36, 37]. Such models are indispensable for validating mechanotransduction findings and accelerating translational discovery.

Despite clear bursts in ‘drug design’ and related clusters, a substantial bench‐to‐bedside gap persists. Current antifibrotic agents (nintedanib and pirfenidone) slow progression but do not directly intercept mechanosensing pathways. Preclinical targets abound—integrins, FAK, YAP/TAZ inhibitors and PIEZO1 modulators—yet clinical translation remains limited by off‐target effects and the ubiquitous expression of mechanosensors [38, 39]. Selective PIEZO1 antagonists and localized delivery strategies (e.g., inhalable nanoparticles) represent promising avenues with narrower therapeutic windows [40]. Overcoming specificity and toxicity challenges constitutes the field's most urgent translational hurdle.

A pivotal emerging frontier identified here is mechano‐immunology. The strong recent burst in ‘immune cells’ and the rising trajectory of Topic 1 (macrophage activation in fibrosis) underscore a conceptual expansion: immune populations actively sense and respond to matrix stiffness, modulating polarization, cytokine release and resolution independently of classical chemokines. Macrophages on stiff substrates adopt proinflammatory M1‐like phenotypes, while compliant matrices favour anti‐inflammatory and pro‐resolving states [41]. Single‐cell and spatial transcriptomics have begun mapping these heterogeneous mechanosensitive niches, revealing stiffness‐dependent metabolic reprogramming and epigenetic changes [42]. This crosstalk explains why inflammation persists in fibrotic lungs despite removal of the initial trigger and positions mechano‐immunology as a high‐yield target for next‐generation immunomodulatory therapies.

The rapid surge in Topic 4 (viral‐associated ARDS, particularly SARS‐CoV‐2) illustrates the field's adaptability to global health crises. Established VILI principles of barrier disruption under excessive strain were swiftly applied to COVID‐19 pathophysiology, where viral infection synergizes with mechanical ventilation to amplify endothelial and epithelial injury. This convergence has accelerated research into combined antiviral–mechanoprotective strategies and reinforced the clinical relevance of stiffness signalling in infectious contexts.

Geographically, a bipolar structure persists, with the United States leading in foundational and high‐centrality contributions and China driving rapid translational expansion post‐2012. Dense intra‐US institutional networks contrast with more limited cross‐national linkages. Strengthening trans‐Pacific and European collaborations will be critical for large‐scale validation studies, diverse patient cohorts and equitable global progress. By integrating CiteSpace‐based citation networks with LDA semantic modelling, this study achieved convergent, multidimensional insights. Keyword bursts flagged mechano‐immunology and advanced models, while LDA quantified the rising dominance of macrophage‐centred and viral topics and the decline of classical overdistension paradigms. This complementary approach enhances robustness beyond traditional bibliometrics.

Several limitations warrant consideration. Reliance on the WoSCC ensures high‐quality data but may exclude relevant engineering, biophysics or materials science literature indexed elsewhere (e.g., Scopus‐exclusive journals). Citation‐based metrics carry temporal lag, potentially under‐representing very recent breakthroughs. The English‐language restriction may overlook valuable non‐English contributions, particularly from emerging research nations. Finally, bibliometric methods capture publication and citation patterns but cannot assess unpublished negative results or ongoing unregistered trials.

In conclusion, pulmonary mechanoregulation has matured from injury prevention to molecular reprogramming of pathological stiffness. Current frontiers converge on mechano‐immunology, biomimetic engineering and multi‐omics integration. Future breakthroughs will likely arise from physiologically faithful models, spatially resolved single‐cell analyses and selective mechanotargeted therapies that safely decouple physical forces from maladaptive remodelling. This bibliometric roadmap equips researchers, clinicians and funders with a strategic overview to prioritize high‐impact directions and foster interdisciplinary collaboration in this vibrant and clinically urgent field.

## 5 Conclusions

This bibliometric study presents the first comprehensive mapping of 25 years of research on mechanotransduction in pulmonary injury. It documents exponential publication growth led by the United States and China and a clear paradigm shift from macroscopic prevention of VILI to molecular analysis of microenvironmental cues, particularly matrix stiffness, YAP/TAZ signalling and mechanosensitive ion channels (e.g., PIEZO1). Citation burst detection and LDA topic modelling converge in identifying current frontiers: declining emphasis on classical mechanical stress (e.g., alveolar overdistension) and rising prominence of mechano‐immunology (especially macrophage activation in fibrosis) and viral‐associated ARDS. Future breakthroughs will likely require integration of high‐fidelity biomimetic models with multi‐omics and single‐cell approaches to develop targeted mechanotherapeutics for fibrotic and inflammatory lung diseases.

## Author Contributions

**Liyan Luo:** conceptualization, methodology, writing – original draft. **Bin Yao:** methodology, software. **Qiqi Ruan:** data curation, investigation. **Yu He:** validation, formal analysis. **Meiyu Zhang:** visualization, resources. **Qing Ai:** investigation, resources, data curation. **Qiuju Wu:** software, formal analysis. **Sile Hu:** methodology, validation. **Ying Zhang:** visualization. **Feng Jiang:** conceptualization, writing – review and editing, supervision. **Yuan Shi:** project administration, supervision, writing – review and editing, conceptualization.

## Funding

This work was supported by the Special Research Fund of the Wu Jieping Medical Foundation (Grant No. 320.6750.2025‐9‐13), the Maternal and Child Nutrition and Health Research Program of the National Center for Women and Children's Health, National Health Commission of the People's Republic of China (Grant No. 2024FYH14), Shanghai Magnolia Talent Program Pujiang Project (Grant No. 25PJD010) and the National Natural Science Foundation of China (Grant No. 82571974).

## Ethics Statement

The authors have nothing to report.

## Consent

The authors have nothing to report.

## Conflicts of Interest

The authors declare no conflicts of interest.

## Supporting information


**Figure S1:** Top 25 references with the strongest citation bursts.

**Figure S2:** Top 25 keywords with the strongest citation bursts.

**Table S1:** Top 10 countries and institutions contributing to mechanoregulation research in lung injury.

**Table S2:** Top 10 authors and co‐cited authors in mechanoregulation research in lung injury.

**Table S3:** Top 10 journals and co‐cited journals in mechanoregulation research in lung injury.

**Table S4:** Top 10 highly co‐cited references in mechanoregulation research in lung injury.

## Data Availability

The datasets generated and analysed during the current study are available from the Web of Science Core Collection (https://www.webofscience.com/). The raw data supporting the conclusions of this article will be made available by the authors, without undue reservation, to any qualified researcher upon reasonable request.
