# Supplementary material for: From Mechanical Cues to Lung Injury: Mapping the Global Landscape and Frontiers of Mechanoregulation Through Bibliometrics and LDA Analysis
Source: J Cell Mol Med. 2026 Sep 25;30(18):e71377. doi: 10.1111/jcmm.71377 (PMC13614878; doi:10.1111/jcmm.71377)
Supplement: Supplementary file 1 [file JCMM-30-e71377-s002.docx]

**Table S3**. Top 10 journals and co-cited journals in mechanoregulation research in lung injury.

| Rank | Journal | Count | IF | JCR | Co-cited Journal | Co-citation | IF | JCR |
| --- | --- | --- | --- | --- | --- | --- | --- | --- |
| 1 | American Journal of Physiology-Lung Cellular and Molecular Physiology | 101 | 3.5 | Q1 | Journal of Biological Chemistry | 4230 | 3.9 | Q2 |
| 2 | American Journal of Respiratory Cell and Molecular Biology | 71 | 5.3 | Q1 | American Journal of Physiology-Lung Cellular and Molecular Physiology | 3722 | 3.5 | Q1 |
| 3 | International Journal of Molecular Sciences | 40 | 4.9 | Q1 | American Journal of Respiratory and Critical Care Medicine | 3709 | 19.4 | Q1 |
| 4 | PLOS One | 39 | 2.6 | Q2 | Journal of Clinical Investigation | 2800 | 13.6 | Q1 |
| 5 | Respiratory Research | 33 | 5.0 | Q1 | American Journal of Respiratory Cell and Molecular Biology | 2723 | 5.3 | Q1 |
| 6 | American Journal of Respiratory and Critical Care Medicine | 32 | 19.4 | Q1 | Proceedings of the National Academy of Sciences of the United States of America | 2528 | 9.1 | Q1 |
| 7 | Scientific Reports | 30 | 3.9 | Q1 | Nature | 1977 | 48.5 | Q1 |
| 8 | Journal of Biological Chemistry | 28 | 3.9 | Q2 | Journal of Cell Biology | 1877 | 6.4 | Q1 |
| 9 | Frontiers in Immunology | 26 | 5.9 | Q1 | American Journal of Pathology | 1836 | 3.6 | Q1 |
| 10 | American Journal of Pathology | 22 | 3.6 | Q1 | Cell | 1826 | 42.5 | Q1 |
