# Supplementary material for: From Mechanical Cues to Lung Injury: Mapping the Global Landscape and Frontiers of Mechanoregulation Through Bibliometrics and LDA Analysis
Source: J Cell Mol Med. 2026 Sep 25;30(18):e71377. doi: 10.1111/jcmm.71377 (PMC13614878; doi:10.1111/jcmm.71377)
Supplement: Supplementary file 2 [file JCMM-30-e71377-s003.docx]

**Table S4**. Top 10 highly co-cited references in mechanoregulation research in lung injury.

| Rank | Co-cited reference | Citations |
| --- | --- | --- |
| 1 | Munger Js, 1999, Cell, V96, P319 | 174 |
| 2 | Liu F, 2015, Am J Physiol-Lung C, V308, Pl344 | 125 |
| 3 | Horan Gs, 2008, Am J Resp Crit Care, V177, P56 | 107 |
| 4 | Wipff Pj, 2007, J Cell Biol, V179, P1311 | 106 |
| 5 | Dupont S, 2011, Nature, V474, P179 | 98 |
| 6 | Ware Lb, 2000, New Engl J Med, V342, P1334 | 96 |
| 7 | Liu F, 2010, J Cell Biol, V190, P693 | 95 |
| 8 | Brower Rg, 2000, New Engl J Med, V342, P1301 | 87 |
| 9 | Henderson Nc, 2013, Nat Med, V19, P1617 | 84 |
| 10 | Hynes Ro, 2002, Cell, V110, P673 | 84 |
