# Supplementary material for: From Mechanical Cues to Lung Injury: Mapping the Global Landscape and Frontiers of Mechanoregulation Through Bibliometrics and LDA Analysis
Source: J Cell Mol Med. 2026 Sep 25;30(18):e71377. doi: 10.1111/jcmm.71377 (PMC13614878; doi:10.1111/jcmm.71377)
Supplement: Supplementary file 3 [file JCMM-30-e71377-s004.docx]

**Table S2**. Top 10 authors and co-cited authors in mechanoregulation research in lung injury.

| Rank | Authors | Count | Co-cited authors | Citations |
| --- | --- | --- | --- | --- |
| 1 | J G Garcia | 27 | B Hinz | 311 |
| 2 | Daniel J Tschumperlin | 24 | G Raghu | 310 |
| 3 | Konstantin G Birukov | 23 | Anna A Birukova | 284 |
| 4 | Anna A Birukova | 22 | Fei Liu | 267 |
| 5 | Dean Sheppard | 20 | Daniel J Tschumperlin | 245 |
| 6 | Yong Zhou | 14 | T A Wynn | 233 |
| 7 | Wolfgang M Kuebler | 14 | J S Munger | 233 |
| 8 | Boris Hinz | 13 | Yong Zhou | 170 |
| 9 | Shelia M Violette | 13 | Richard O Hynes | 157 |
| 10 | Simon J F Macdonald | 13 | Michael A Matthay | 153 |
