# Supplementary material for: From Mechanical Cues to Lung Injury: Mapping the Global Landscape and Frontiers of Mechanoregulation Through Bibliometrics and LDA Analysis
Source: J Cell Mol Med. 2026 Sep 25;30(18):e71377. doi: 10.1111/jcmm.71377 (PMC13614878; doi:10.1111/jcmm.71377)
Supplement: Supplementary file 4 [file JCMM-30-e71377-s005.docx]

**Table S1**. Top 10 countries and institutions contributing to mechanoregulation research in lung injury.

| Rank | Country | Count | Institution | Count |
| --- | --- | --- | --- | --- |
| 1 | United States | 884 | University of California, San Francisco (United States) | 83 |
| 2 | China | 501 | University of Toronto (Canada) | 54 |
| 3 | Germany | 148 | University of Illinois (United States) | 50 |
| 4 | United Kingdom | 141 | University of Michigan (United States) | 46 |
| 5 | Canada | 116 | University of Chicago (United States) | 44 |
| 6 | Japan | 111 | Shanghai Jiao Tong University (China) | 39 |
| 7 | Italy | 55 | Harvard Medical School (United States) | 37 |
| 8 | France | 51 | Harvard University (United States) | 34 |
| 9 | Netherlands | 46 | Mayo Clinic (United States) | 33 |
| 10 | South Korea | 39 | University of Alabama at Birmingham (United Kingdom) | 33 |
